# Open-Set Recognition of Fire Smoke from Four-Channel Optical Smoke Detector

**DOI:** 10.3390/s26206606

**Published:** 2026-10-19

**Authors:** Liangbo Li, Ming Zhu, Mengxue Lin, Jiahao Gao, Linsen Zhao, Maosen Wang

**Affiliations:** 1 Hubei Key Laboratory of Smart Internet Technology, School of Electronic Information and Communications, Huazhong University of Science and Technology, Wuhan 430074, China; 2 School of Electronic Engineering, Naval University of Engineering, Wuhan 430033, China

**Keywords:** fire detection, fire smoke, interference aerosol, open-set recognition, optical detector

## Abstract

**Highlights:**

**What are the main findings?**

- Design an open-set classification model to accurately distinguish whether aerosols not present in the training dataset are actual fire smoke or interference aerosols.
- Develop a 4-channel optical scattering fire smoke detector capable of identifying interference sources.

**What is the implication of the main finding?**

- Based on class prototype distances, the model predicts the probability that arbitrary aerosols belong to fire smoke or interference aerosols, providing quantifiable confidence for distinguishing unknown aerosols in real-world fire detection.

**Abstract:**

In fire smoke detection, interference aerosols frequently cause false alarms for photoelectric smoke detectors. Conventional closed-set classification models trained on limited samples cannot reliably handle unknown aerosol types, which further leads to false and missed alarms. To address this issue, we propose FireOSR-Net, an open-set recognition network for fire smoke identification using the signals of a four-channel optical scattering detector, which integrates three modules. First, Conv1d-LSTM is used to extract features from dual-wavelength, dual-angle scattered signals. Next, the out-of-distribution detection module constructs an open boundary between known and unknown aerosols and generates pseudo-unknown features to enhance the detection capability for out-of-domain samples. Finally, the distance of all scattered intensity signals to both the fire and the non-fire domain is calculated, and fire and non-fire proximity scores are output to assess their risk propensity. Experiments are performed on eight-class aerosol data acquired with our self-developed four-channel detector, including six known and two unknown test categories. It achieves a peak F1-score of 0.95 on known samples, while unknown smoldering-wire smoke and oil fumes reach 96.29% fire proximity and 89.68% non-fire proximity correspondingly. Such results demonstrate FireOSR-Net’s capacity for aerosol classification, out-of-domain detection and risk evaluation towards interpretable low-false-alarm fire sensing.

## 1. Introduction

Early fire detection is a key technology for smart fire safety systems, industrial safety management, and complex facility monitoring. Modern buildings feature more open layouts, and monitoring scenarios are becoming increasingly complex. This has led to changes in the evaluation criteria for fire detection. Systems now focus not only on whether an alarm is triggered but also place higher demands on detection real-time performance, interference resistance, identification robustness, and decision explainability [1,2,3,4,5]. Among the various early-detection technologies, optoelectronic detection solutions based on aerosol scattering characteristics offer distinct advantages. With fast response times, low hardware costs, and ease of embedded deployment, this approach has become the mainstream research direction for fire smoke identification [6,7,8,9,10]. The multi-wavelength and multi-angle light scattering mechanism can extract aerosol features from multiple dimensions, including particle size distribution, and refractive index characteristics. This mechanism can effectively distinguish between fire smoke and non-fire interference aerosols, providing a rich data foundation for high-precision identification [11,12,13,14,15].

To date, scholars have conducted extensive research on the discrimination between fire smoke and interference aerosols. Chaudhry et al. [16] used multiband light attenuation characteristics to identify combusting materials, demonstrating the discriminatory potential of multi-wavelength optical features. However, this method relies on broad-spectrum measurement equipment, which is structurally complex and difficult to adapt to the miniaturization and integration requirements of commercial detectors. Gottuk et al. [17] developed a composite alarm algorithm by fusing multi-sensor features, effectively improving the reliability of fire detection. Loepfe et al. [18] and Keller et al. [19] systematically studied the optical properties and online response patterns of fire and non-fire aerosols, providing theoretical support for multi-channel light scattering detection technology. Dinaburg et al. [20] comprehensively summarized the sources of detector false alarms. Research indicates that oil fumes, ambient water vapor, and airborne dust are the primary interference factors affecting the operational stability of the equipment. Zheng et al. [15] employed the asymmetric ratio of two-wavelength, dual-angle scattering to distinguish between fire and interference aerosols. While this method performs well in identifying conventional samples such as black smoke, white smoke, and dust, its ability to distinguish complex interference samples with high feature similarity is insufficient, leaving significant optimization potential.

Dual-wavelength and multi-angle light scattering sensing technologies have been continuously optimized for fire detection. Deng et al. [13] achieved optimization of aerosol concentration inversion and refractive index compensation based on the dual-wavelength scattering principle. Wang et al. [14] utilized light scattering characteristics to accurately estimate the Sauter mean particle diameter of fire smoke. Xia [3] et al. demonstrated, through three-wavelength and dual-wavelength measurement systems, respectively, the positive role of multi-channel scattering information in improving recognition accuracy. Li et al. [4] effectively reduced the system’s false alarm rate by combining scattering and extinction measurements. Kang et al. [1] demonstrated through broad-spectrum scattering simulations that fire smoke and typical interference aerosols exhibit distinguishable differences in their multiband responses. In summary, aerosol recognition methods that integrate multidimensional sensing information with deep learning algorithms have become a research focus in the field of fire detection.

Existing recognition methods are based on closed-set classification theory, which assumes that the classes of all test samples are already included in the training dataset. This idealized assumption is difficult to apply to real-world, complex fire monitoring scenarios. Factors such as new combustion materials, environmental interference, and scene variations can all generate out-of-domain samples not present in the training dataset. Traditional closed-set classifiers force unknown samples into a known category. This mechanism is highly prone to causing false positives and false negatives [21,22,23,24]. In fire protection engineering, this flaw poses significant safety risks. If unknown interference is misclassified as a fire, it will undermine the reliability of the equipment. Conversely, if unknown fire smoke is misclassified as interference, the opportunity for early fire suppression may be missed, leading to serious safety incidents. Therefore, interference-resistant recognition of fire smoke cannot simply be equated with conventional multi-class classification tasks. This task is essentially an open-set recognition problem, encompassing requirements such as accurate classification of known categories, detection of out-of-domain unknown samples, and risk prediction. Open-set recognition provides reasonable rejection capabilities for unseen samples while ensuring the recognition accuracy of known samples [25,26,27,28]. However, merely identifying unknown samples is still insufficient to meet the practical needs of firefighting decision-making.

To address the above issues, we propose an open-set recognition network for interference-resistant fire smoke recognition. This model includes modules for feature extraction, open-set detection, and fire proximity score calculation. The model first extracts deep features from the raw time-series data of fire smoke and interference aerosols. Next, it determines whether an input sample belongs to a known class or is an unknown aerosol outside the domain. When a sample is identified as known, it is fed into a known-class classifier to output the specific aerosol class. Simultaneously, the model calculates the distance between the test sample and known fire smoke and non-fire aerosols to quantitatively determine the that the sample belongs to the fire or non-fire class. Unlike traditional models that only optimize the accuracy of closed-set recognition, the network designed in this work focuses on the needs of real-world monitoring scenarios and can prevent the model from forcibly outputting an incorrect class when presented with unknown smoke or interference aerosols. Furthermore, this work establishes a joint analysis mechanism combining the proximity ratio and class confidence to quantify the risk of unknown samples. The proximity ratio characterizes the relative distance of an unknown sample to both fire and non-fire conditions, while class confidence measures the reliability of the classification result. The combined use of these two metrics enhances the robustness and interpretability of the classification results. The main innovations of this study are summarized as follows:

(1) Design an open-set classification model to accurately distinguish whether aerosols not present in the training dataset are actual fire smoke or interference aerosols.
(2) Predict the probability that arbitrary aerosols belong to fire smoke or interference aerosols based on class prototype distances.
(3) Develop a 4-channel optical scattering fire smoke detector capable of identifying interference sources.

The remaining parts of this paper are organized as follows. Section 2 describes the optical scattering principle, detector configuration, dataset construction, preprocessing procedure, and network architecture. Section 3 describes the experimental parameter settings, presents the experimental results, and conducts an in-depth analysis. Section 4 summarizes the conclusions of this study and discusses its limitations and prospects for future research.

## 2. Materials and Methods

### 2.1. Optical Scattering Principle of Fire Smoke and Interference Aerosols

Fire smoke and interference aerosols are essentially polydisperse aerosol systems composed of a large number of microscopic particles. When incident light interacts with suspended particles, its propagation direction and energy distribution change, resulting in omnidirectional scattered light. The scattering response of particles to incident light is primarily determined by a combination of particle size, refractive index, particle morphology, and concentration. When the particle size is on the same order of magnitude as the wavelength of the incident light, Mie scattering theory can adequately describe the angular scattering distribution [1,2,18]. Given a particle diameter of *d* and an incident wavelength of *λ*, the dimensionless particle size is defined as

(1) $$x=\frac{\mathrm{\pi d}}{\lambda }$$

For a single spherical particle, the scattering intensity *I_S_* depends not only on *x*, but also on the scattering angle *θ* and the scattering amplitude functions *S*_1_(*x*,*θ*) and *S*_2_(*x*,*θ*):

(2) $$I_{S}\left(\lambda ,x,\theta \right)=\frac{\lambda^{2}}{4\pi^{2}r^{2}}\left|\frac{S_{1}\left(x,\theta \right)+S_{2}\left(x,\theta \right)}{2}\right|I_{0}$$

where *I*_0_ is the initial intensity of the incident light, and *r* is the observation distance.

In actual detection, isolated individual particles do not exist. Both fire smoke and interference aerosols consist of a vast number of particles of varying sizes, and the total scattered light intensity of an aerosol particle ensemble is the linear sum of the scattered light intensities of all individual particles. The total scattered light intensity of an aerosol ensemble consisting of *N* particles can be expressed as

(3) $$I_{\det}\left(\lambda ,\theta \right)=\sum_{i=1}^{N}I_{S}\left(\lambda ,x,\theta \right)N\left(d\right)$$

where *I_S_*(*λ*,*x*,*θ*) represents the independent scattered light intensity of the *i_th_* aerosol particle, and *N*(*d*) is the aerosol particle size distribution function, which describes the proportion of particles of different sizes.

When light emitted by the LED sources interacts with aerosol particles, the scattering responses corresponding to different wavelengths and observation angles can be simultaneously detected by photodetectors, thereby constructing a scattering observation vector with multidimensional characteristics. Forward scattering signals are highly sensitive to changes in aerosol particle concentration, while wide-angle backscattering signals can effectively characterize differences in particle microstructure and composition [9,11]. Based on the optical scattering mechanisms, we designed and developed a four-channel fire photodetector that combines dual-wavelength, dual-angle, multidimensional scattering characteristics for the precise identification of fire smoke.

### 2.2. Four-Channel Photoelectric Smoke Detector

**Figure 1:**
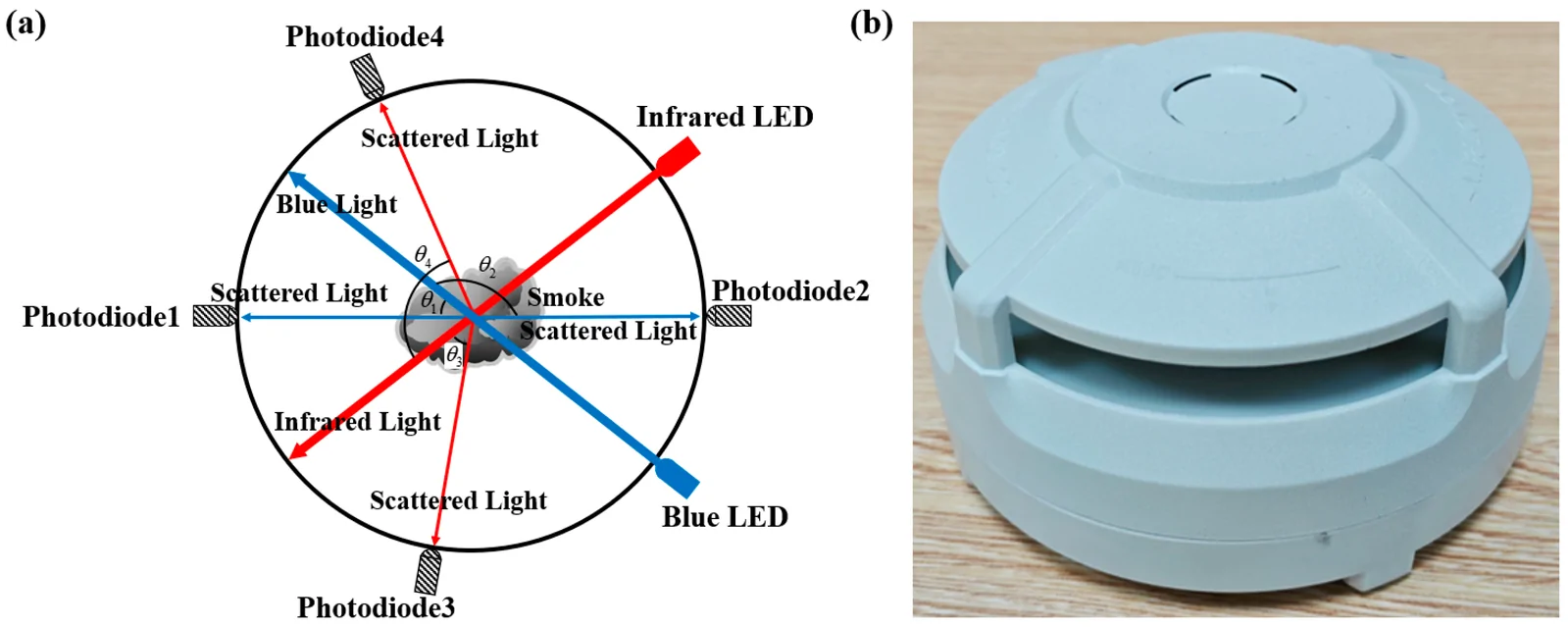
Schematic diagram and hardware implementation of the four-channel optical fire smoke detector. (**a**) Schematic diagram of the detection principle, (**b**) Photograph of the detector.

The dual-wavelength, dual-angle, four-channel fire smoke detector developed in this study is shown in Figure 1. Using combined measurements from multiple optical channels, the detector captures the scattering response of aerosol under different wavelength and scattering angle conditions. Figure 1a illustrates the detector’s operating principle, while Figure 1b shows a photograph of the developed detector’s hardware. The detector employs a 450 nm blue LED and a 950 nm near-infrared LED as dual-wavelength light sources, and utilizes four photodetectors to respectively receive the scattered light intensity under different observation conditions. The four detection channels correspond, in order, to 450 nm/40°, 450 nm/140°, 950 nm/40°, and 950 nm/100°. Combining optical information from both wavelength and angle dimensions enables the detector to characterize aerosol properties, such as particle size, microstructure, and concentration variations, based on the measured scattered light intensity.

Scattering channels at different wavelengths respond differently to particle size [29]. The 450 nm short-wavelength channel is more sensitive to scattering changes in small-diameter particles and is suitable for detecting fire smoke particles. Since particles generated in the early stages of a fire are smaller in diameter, they produce more pronounced changes in scattering response when illuminated by short wavelengths. The 950 nm near-infrared channel responds well to larger particles and is more effective at identifying larger dust particles and water vapor. Dual-wavelength smoke detectors can cover a wider range of particle sizes and enhance the ability to detect aerosols with different size distributions.

The scattering angle has a stronger influence on the morphological characteristics of particles [29]. The wide-angle scattering channels at 100° and 140° are more sensitive to differences in particle morphology, refractive index, and variations in the scattering phase function. Fire smoke particles typically exhibit irregular shapes and complex agglomeration structures, whereas oil fumes, water vapor, and dust particles display distinct characteristics in terms of particle size distribution and morphology. Wide-angle scattering responses amplify these structural differences, enabling different aerosols to exhibit more distinctive response characteristics under multi-angle observation. Therefore, the four-channel detector developed in this study simultaneously captures the particle size distribution, morphology, and concentration of particle groups through the combined response of multidimensional scattered light intensity, thereby improving the reliability of fire smoke identification in complex environments.

Furthermore, combustion is a continuously evolving physicochemical process. In the early stages of a fire, combustible materials undergo continuous pyrolysis and oxidation. During this process, smoke particles are constantly generated, coalesce, and disperse into the detection area. Particle concentration, size distribution, and microstructure continue to change over time. The multi-channel scattering signals collected by the detector also undergo dynamic changes accordingly. Therefore, the difference between fire smoke and interference aerosols lies not only in the scattering intensity at a single moment but also in the rate of change in the scattering intensity. To more fully utilize the dynamic scattering characteristics of aerosols, this work introduces multichannel scattering data with temporal evolution characteristics and uses the multichannel scattering time-series data **I_det_** as model input, as shown in Equation (4). **I_det_** characterizes particle size and morphological features through differences in multidimensional optical responses from dual-wavelength, dual-angle detector, while simultaneously characterizing the generation and diffusion processes of fire smoke or interference aerosols through trends in the temporal dimension.

**I_det_** = [*I*_det_(450, 40°), *I*_det_(450, 140°), *I*_det_(950, 40°), *I*_det_(950, 100°)] (4)

The core control unit of the detector is a 32-bit ARM microcontroller (STM32F103, Alientek, Guangzhou, China), which governs the entire sensing procedure, including LED lighting scheduling, signal sampling, data processing, and state judgment. The system employs a two-wire bus communication protocol to realize duplex data transmission and synchronous power supply between the detector and the upper controller. This integrated bus design simplifies on-site wiring, reduces structural complexity, and lowers overall system deployment costs, while supporting multi-device cascading such that a single controller can stably manage multiple distributed detectors. In terms of power supply configuration, the main control chip operates at 3.3 V, whereas the optical detection module works at a rated voltage of 5 V. During measurement, the microcontroller precisely controls the turn-on timing and working period of the dual-wavelength LEDs to generate stable and periodic optical excitation signals. The photodiodes deployed in each detection channel convert real-time aerosol scattered light intensity into analog electrical signals. These raw analog signals are conditioned via analog amplification and filtering circuits to suppress high-frequency noise and interference and are subsequently transmitted to the internal ADC module of the STM32F103 chip for high-precision analog-to-digital conversion. After digitization, the microcontroller processes the multi-channel time-series scattering data, evaluates the current aerosol environment state, and generates corresponding alarm flag information. Finally, all detection results and status signals are uploaded to the upper controller through the two-wire bus, forming a complete closed-loop sensing and transmission workflow.

### 2.3. Fire Smoke and Interference Aerosol Dataset

**Figure 2:**
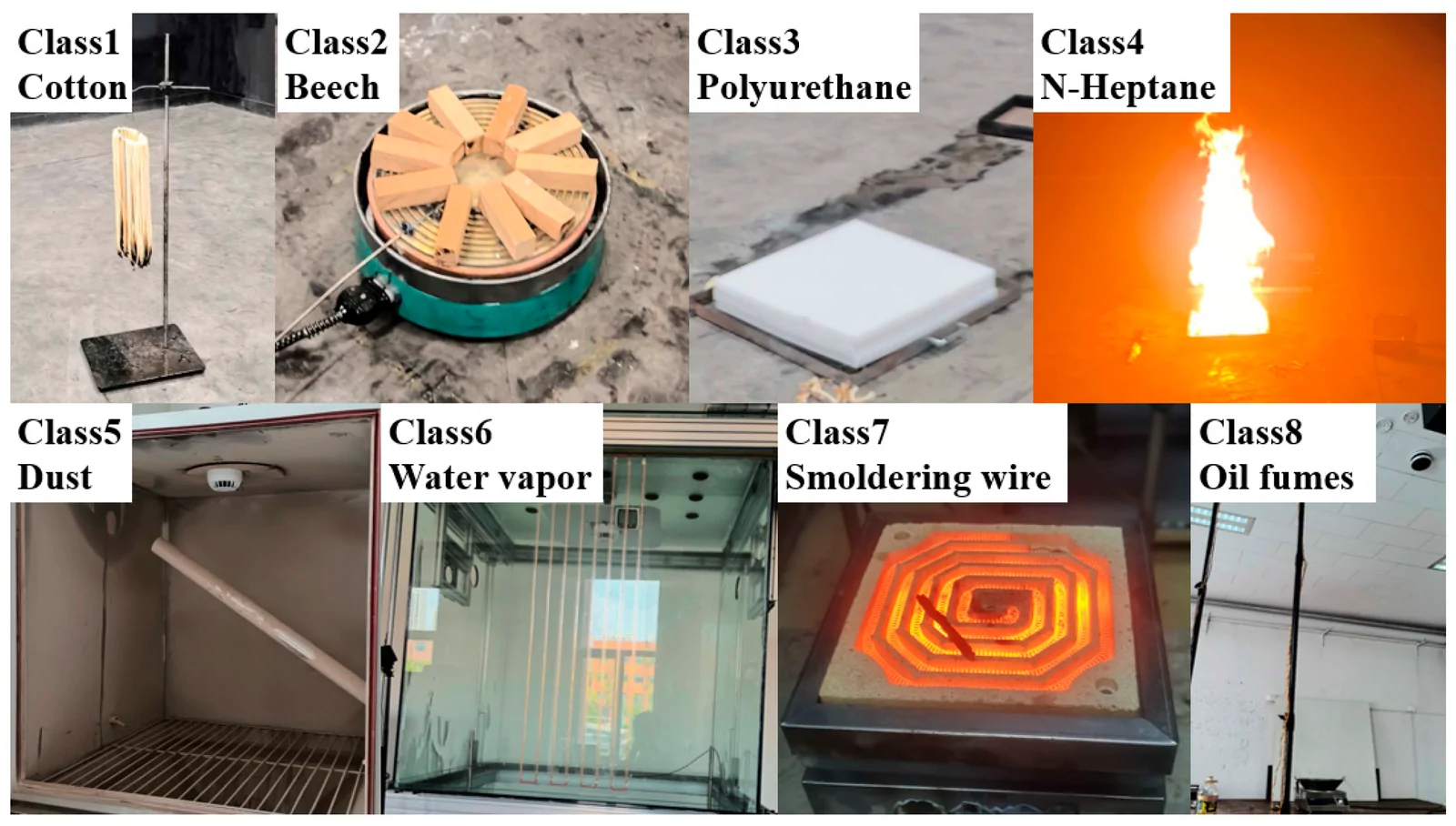
Experimental platforms for collecting fire-smoke and interference aerosol samples.

This dataset consists of measurement results obtained from our four-channel fire smoke detector during testing on an integrated combustion platform, in a water vapor chamber, and in a dust chamber, as shown in Figure 2. It includes a total of eight types of aerosol samples. Among these, five types represent typical fire smoke: beech pyrolysis, smoldering cotton, open flame polyurethane, open flame N-Heptane, and smoldering electrical wiring. The other three sample types are typical interference aerosols: water vapor, dust, and oil fumes. These samples cover smoldering solid fuels, open flames from liquid fuels, and common non-fire interference sources, thereby comprehensively encompassing the primary aerosol types encountered in real-world fire detection scenarios.

According to the open-set classification task, smoke from smoldering electrical wires and oil fumes were designated as unknown classes, used solely for model evaluation during the testing phase. The remaining four classes of fire smoke and two classes of interference aerosols are designated as known classes for model training and closed-set testing. This classification scheme allows for the evaluation of the model’s classification performance within the scope of the training classes, while also assessing its ability to distinguish between fire smoke and interference aerosols not included in the training data.

The detector outputs signal data from four channels at each time point, denoted as *I*_0_, *I*_1_, *I*_2_, and *I*_3_, respectively. The raw measurement data is stored in CSV files. To reduce the impact of measurement noise on time-series modeling and to preserve the physical trends of the signals as much as possible, the four-channel sequences are first subjected to preliminary Gaussian filtering. Subsequently, the valid response interval is determined based on the L2-norm of the four-channel vector, and the valid signal is divided into three phases: early, mid, and late. Let the four-channel signal vector at time step *t* be denoted as **s_t_** = [*s_t_*_1_, *s_t_*_2_, *s_t_*_3_, *s_t_*_4_], then its amplitude is defined as

(5) $${\left\|s_{t}\right\|}_{2}=\sqrt{\sum_{i=1}^{4}s_{\mathrm{ti}}^{2}}$$

When ${\left\|s_{t}\right\|}_{2}$ exceeds a preset threshold, that time step is considered a valid scattering response. The first valid response point is taken as the starting point of the aerosol signal, and the point of maximum intensity is taken as the end point of the rising phase. Subsequently, the rising phase is divided into early and mid-stages according to a fixed ratio, while the signal following the peak is classified as the late stage. Given the requirement for rapid response in early fire warning systems, the early-stage signal was used to construct the recognition samples in the main experiments. This stage contains critical scattering information from the initial formation and rapid changes in aerosols, better reflecting the model’s recognition capability under early-stage conditions with weak responses. During sample generation, the early-stage signal is segmented using a sliding window with a window length of 10 and a step size of 2. Let the valid sequence be **s** = [**s_1_**, **s_2_**, …, **s_L_**], then, the *n_th_* sliding window sample is represented as

**X_n_** = [**s_1+nΔ_**, **s_2+nΔ_**, …, **s_T+nΔ_**] ∈ *ℝ^T^*^×4^ (6)

where *T* represents the window length, and Δ represents the sliding step size. After sliding window processing, a continuous scattering sequence is converted into several short-time samples, each length of 10 and 4-dimensional. This processing method improves the utilization efficiency of the measured data and enables the model to focus on local dynamic changes within the rising phase. For early-stage fire smoke detection, these short-term dynamic features help characterize the differences among various aerosols in terms of their growth rates and channel response relationships.

### 2.4. FireOSR-Net: Fire Open-Set Recognition Net

#### 2.4.1. Overall Framework

**Figure 3:**
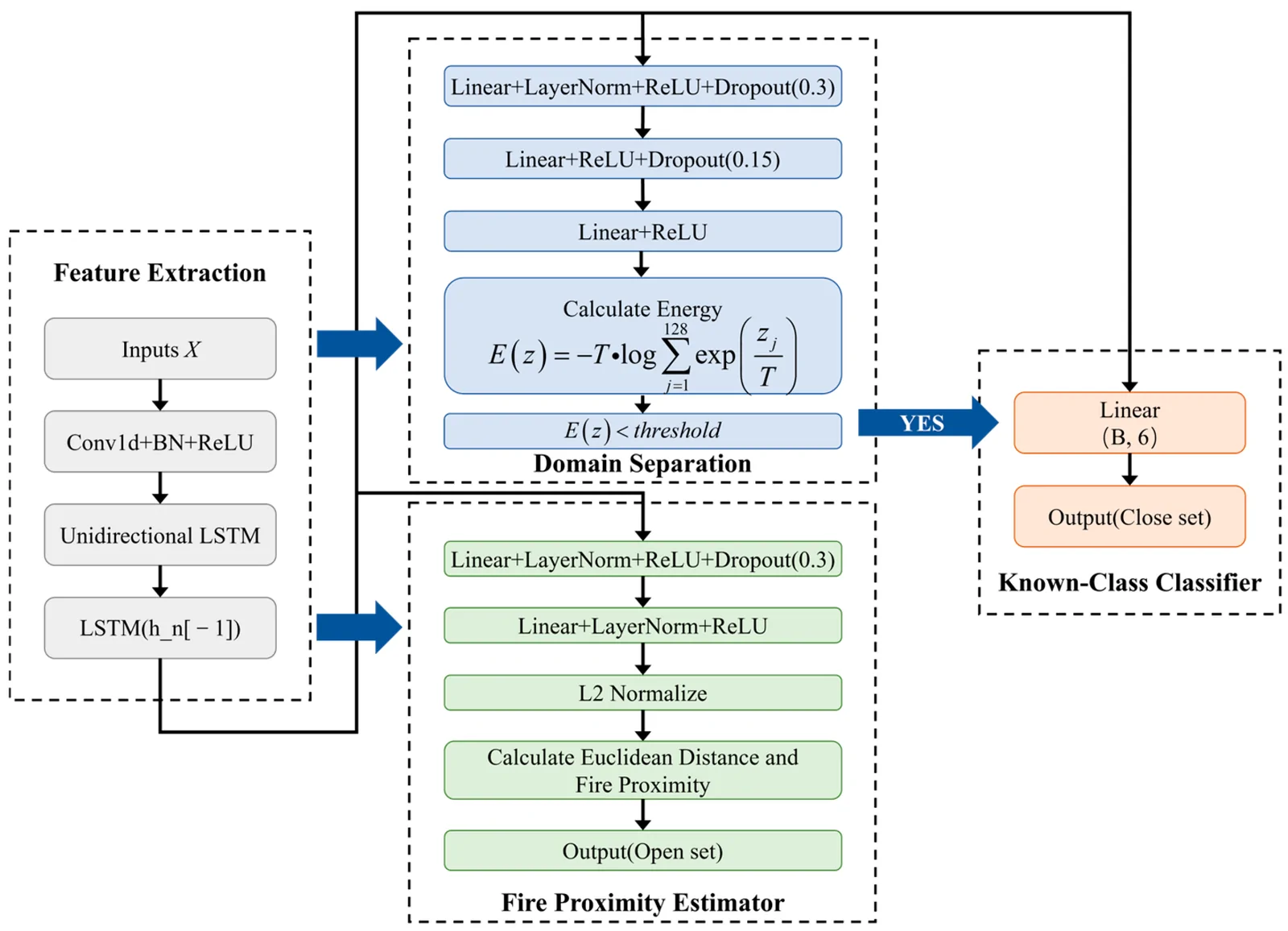
The algorithmic workflow framework of the Fire Open-Set Recognition Net.

The method proposed in this study consists of three main components: a closed-set classification network module, an energy-based out-of-distribution detection module, and a prototype-metric-based fire risk prediction module. The core idea is to first construct representations of known classes through supervised learning, then establish an open boundary between known and unknown classes using an energy-based mechanism, and finally predict the likelihood of a sample being a fire or non-fire event in the prototype space. Figure 3 shows the algorithmic workflow framework of the Fire Open-Set Recognition Net (FireOSR-Net).

#### 2.4.2. Closed-Set Classification Network

A lightweight Conv1d-LSTM closed-set classification network is used for the task of classifying aerosols of known types. The network consists of a one-dimensional convolutional layer, batch normalization, a ReLU activation function, a unidirectional LSTM, and a linear classification layer. The closed-set training loss function uses cross-entropy loss:

(7) $$L_{\mathrm{ce}}=-\sum_{k=1}^{c}y_{k}\log p_{k}$$

where $p_{k}=\frac{e^{z_{k}}}{\sum_{j}e^{z_{j}}}$, and *c* represents the total number of predefined known aerosol categories used for model training. *y_k_* is the one-hot encoded ground-truth label corresponding to the *k_th_* known class. *p_k_* denotes the model-output posterior probability that the input sample belongs to the *k_th_* known aerosol category. This loss constrains the model to learn discriminative feature representations for known aerosol samples during the training phase. The purpose of this stage is to establish a basic discriminative capability for distinguishing between known smoke and interference aerosol, while also providing stable intermediate features for subsequent open-set boundary learning.

#### 2.4.3. Energy-Based Out-of-Distribution Detection Module

An energy-based out-of-distribution detection module is used to distinguish between known and unknown classes of aerosols to be measured. In this process, the closed-set classification network is frozen and serves solely as a feature extractor. Intermediate features **f** = *ϕ*(**x**) from the LSTM are extracted using a forward feature hook and then fed into an energy encoder based on fully connected mappings to obtain the embedding vector **z_d_**:

**z_d_** = *g_ood_*(**f**;Θ_ood_) (8)

where *g_ood_*(·) denotes the energy mapping network in the out-of-distribution detection module, and *Θ_ood_* represents its learnable parameters. In this embedding space, this paper employs an energy function for open-set modeling:

(9) $$E\left(x\right)=-T\log\sum_{i}e^{\frac{z_{\mathrm{di}}}{T}}$$

where *T* is the temperature parameter. The higher the energy, the more the samples tend to deviate from the distribution of the known domain. To ensure that known and unknown samples form a stable separation in energy space, energy constraints are applied to the known and unknown samples, respectively, so that the energy of the former is concentrated in a lower region, while the energy of the latter is pushed toward a higher region. To improve the open boundary’s ability to extrapolate to unseen sources of interference, a pseudo-unknown feature generator is further introduced. This module generates pseudo-out-of-domain features from random noise. Its primary function is to expand the coverage of the unknown space and reduce the risk of overfitting by the open boundary to a small number of true unknown samples.

Unlike empirically fixed thresholds, the decision threshold for energy-based OOD detection is statistically calibrated based on the energy distribution of validation samples after model training convergence, which ensures stable and generalized classification boundaries. Specifically, we calculate the 95*_th_* percentile energy value of known-class samples $E_{\mathrm{known}}^{95}$ and the 5*_th_* percentile energy value of unknown-class samples $E_{\mathrm{unknown}}^{5}$ on the validation set. The midpoint of these two percentile values is defined as the final decision threshold *τ*. This percentile-based calibration strategy effectively balances the classification accuracy of known samples and the detection capability of unknown samples, avoiding subjective threshold setting and improving experimental reproducibility.

During the inference phase, the model calculates the energy score *E*(*z*) for each input time-series sample and performs hierarchical discrimination based on the predefined threshold *τ*. If the calculated energy *E*(*z*) is lower than *τ*, the sample is determined as a known aerosol category. The extracted shared feature is subsequently imported into the closed-set classifier to obtain the specific aerosol category. By contrast, if *E*(*z*) > *τ*, the sample is identified as an unknown out-of-distribution sample. In this case, the feature will not be sent to the closed-set classifier to avoid incorrect known-category classification. Instead, the output of the fire probability calculation module is adopted to further determine whether the unknown sample belongs to the fire smoke domain or the non-fire interference aerosol domain.

#### 2.4.4. Unknown-Sample Semantic Interpretation Based on Proximity Ratio and Class Confidence

In order to not only identify unknown classes after open-set detection but also to further perform a risk analysis on unknown aerosol samples to determine whether they are more likely to be fire-related or interference aerosols, a prototype-space-based module for fire semantic interpretation of unknown samples was designed. First, the minimum distances from the unknown sample to the fire domain and the non-fire domain are calculated and denoted as *d_fire_* and *d_non_*_−*fire*_, respectively. Based on these, the fire proximity ratio and non-fire proximity ratio are defined as follows:

(10) $$P_{\mathrm{fire}}=\frac{d_{\mathrm{non}-\mathrm{fire}}}{d_{\mathrm{fire}}+d_{\mathrm{non}-\mathrm{fire}}},P_{\mathrm{non}-\mathrm{fire}}=\frac{d_{\mathrm{fire}}}{d_{\mathrm{fire}}+d_{\mathrm{non}-\mathrm{fire}}}$$

Although relying only on the proximity ratio can reflect a sample’s bias relative to the two major domains, the proximity ratio may be affected by outlier noise when a sample is distant from both domains. To address this, this work further introduces category confidence. Let ${\left\{d_{c}\right\}}_{c=1}^{C}$ denote the distances from a sample to all known prototypes; then, a distance-based soft confidence distribution can be constructed as follows:

(11) $$q_{c}=\frac{e^{-\omega d_{c}}}{\sum_{j=1}^{C}e^{-\omega d_{j}}}$$

where *ω* is the distance scaling factor. The class confidence of a sample is defined as

(12) $$C_{\max}=\underset{c}{\max}q_{c}$$

If *C*_max_ is large, it indicates that although the sample is classified as an open sample, it still exhibits strong consistency with a particular category or semantic domain in the prototype space. Conversely, if *C*_max_ is small, it suggests that the sample is located at the edge of the prototype space or in a transitional zone between domains, requiring greater caution in interpreting the risk. Taking into account both the proximity ratio and category confidence, the fire risk score for an unknown sample can be conceptualized as follows:

*S_fire_* = *P_fire_* × *C*_max_ (13)

This definition comprehensively considers both the proximity ratio, which reflects directionality, and the category confidence, which reflects reliability. Combining these two factors, the model can not only determine whether the aerosol to be tested is closer to fire smoke or to interference aerosols, but also provide a confidence level for the determination. This design extends open-set detection by providing a fire-related tendency estimate for unknown samples.

## 3. Experiments and Discussion

In this work, a multi-category aerosol dataset covering eight typical aerosol and fire-smoke types was established, including smoldering cotton smoke, beech smoke, polyurethane smoke, n-heptane smoke, dust, water vapor, oil fumes, and wire-smoldering smoke. A total of 40, 40, 40, 40, 120, 120, 40, and 80 independent physical experiments were conducted for the above eight aerosol categories, respectively. Specifically, the four fire smoke types, dust, and water vapor were defined as known categories for model training and closed-set evaluation. In contrast, wire-smoldering smoke and oil fumes were designated as unknown fire and unknown non-fire categories, respectively, to support open-set performance evaluation.

To ensure standardized data processing and rigorous model evaluation, the original dataset was partitioned into training, validation, and test subsets at an 8:1:1 ratio based on independent physical experiment units. Subsequently, sliding window segmentation was implemented for each subset with a window length of 20 and a stride of 2, leading to a 90% overlap between adjacent windows. Given the variable duration of signal recordings across individual physical experiments, the number of segmented windows varied for each experiment, which resulted in a slight deviation of the final window quantity ratio of the three subsets from the initial 8:1:1 partition ratio. The number of segmented window instances for smoldering cotton smoke, beech smoke, polyurethane smoke, n-heptane smoke, dust, water vapor, oil fumes, and wire-smoldering smoke was 9927, 6838, 4992, 4553, 4035, 3923, 4973, and 6053, respectively, with a total of 45,294 valid instances obtained in this study. To eliminate data leakage, all window samples derived from a single physical experiment were strictly assigned to one identical subset.

All model training and numerical validation experiments in this work were performed on a desktop computer equipped with an Intel Core i7-10510U CPU (1.80–2.30 GHz) using PyCharm Community Edition 2020.1. The closed-set classification network was trained for 300 epochs with a batch size of 64 and an initial learning rate of 0.001. The energy-based out-of-distribution detection module was trained for 50 epochs, with an embedding dimension of 128, a hidden layer dimension of 256, and 64 pseudo-unknown samples generated per training batch. Similarly, the prototype-based metric learning module was trained for 50 epochs, adopting an embedding dimension of 128, a prototype scaling factor of 10.0, and a domain separation margin of 1.2. The Adam optimizer was utilized for the entire training process of all modules.

### 3.1. Closed-Set Classification Results

**Figure 4:**
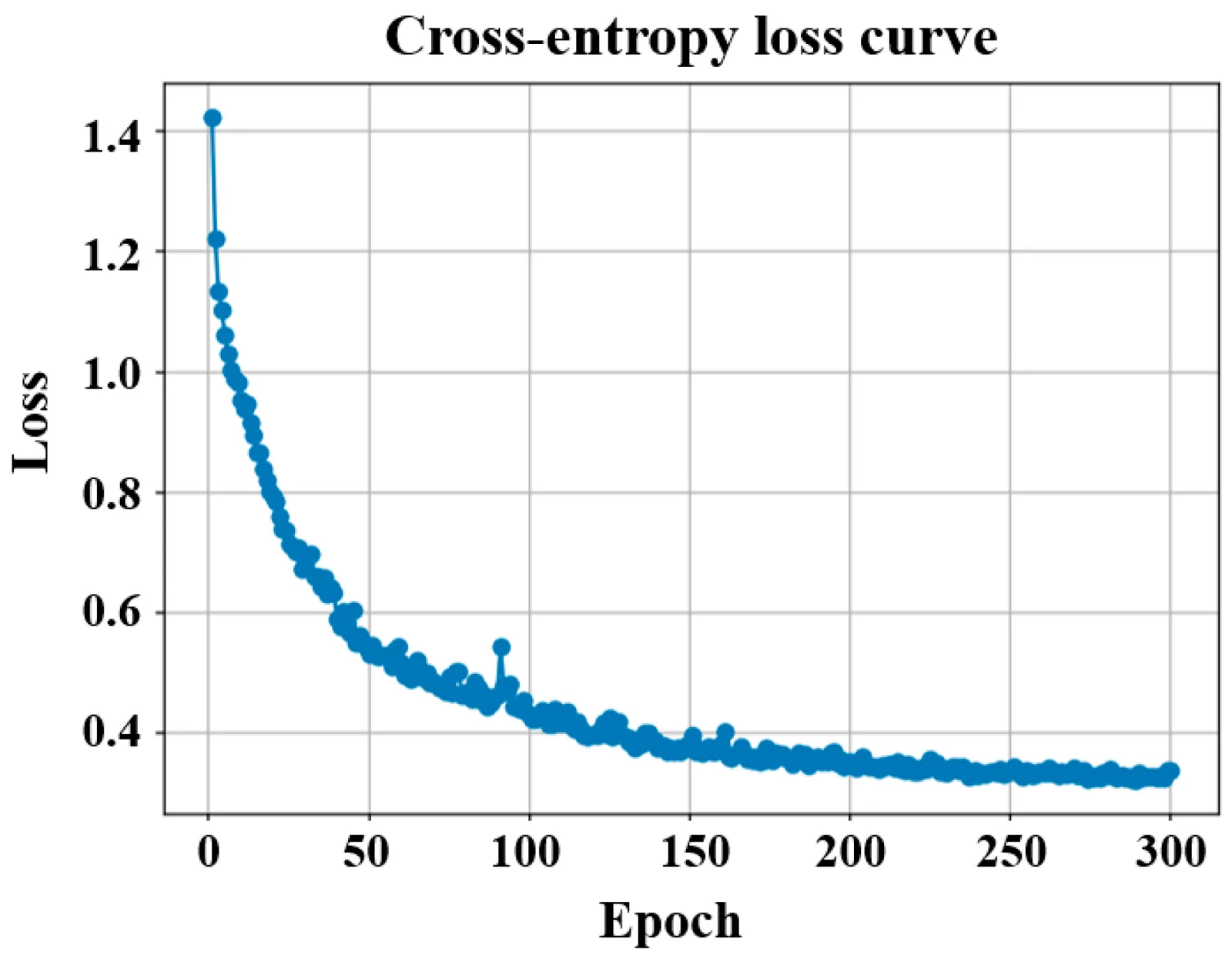
Cross-entropy loss curve of the closed-set classification network during supervised training.

To evaluate the training convergence characteristics and feature learning capabilities of the Conv1d-LSTM temporal backbone network, we plotted the cross-entropy loss curve during the supervised training phase of the backbone network, as shown in Figure 4. During the initial training phase, the network weights were randomly initialized, and the initial loss remained at a relatively high value. The loss decreased rapidly during the first 50 epochs, indicating that the model quickly captured the distinguishing features among the temporal signals of aerosol scattering from different categories, thereby completing the basic feature learning. As the number of training iterations continued to increase, the loss function continued to decrease overall. After approximately 200 epochs, the curve leveled off and stabilized in the range of 0.20–0.25, indicating that the model had reached a state of convergence. This demonstrates that the Conv1d-LSTM temporal classification network is capable of stably learning the discriminative structures within four-channel scattering time series, and that the model maintains good optimization stability under complex multi-class aerosol scattering patterns.

We evaluated the closed-set classification performance of the trained Conv1d-LSTM backbone network using a total of 34,268 test samples. The detailed results for precision, recall, and F1-score across various aerosol sample categories are shown in Table 1. Overall, the model achieved excellent recognition performance for all six known aerosol categories, with F1-scores exceeding 0.80 across all categories. These results indicate that the backbone network can extract discriminative temporal features from four-channel scattering signals.

Specifically, the precision, recall, and F1-score for smoldering cotton smoke were 0.95, 0.91, and 0.93, respectively. These high classification metrics indicate that the temporal scattering characteristics of this class of smoldering smoke are sufficiently distinguishable, resulting in a low proportion of misclassified samples and the ability to detect the vast majority of cotton smoldering samples. As for pyrolysis beech smoke, the recall rate reaches 0.94 and the precision rate is 0.84. The high recall rate indicates that the model is less prone to missing this class of smoke, while the lower precision rate stems from overlapping features in the early scattering signals of various aerosol classes, resulting in some samples from other categories being incorrectly classified as beech pyrolysis smoke. The precision and recall rates for both polyurethane open-flame combustion and N-Heptane open-flame combustion products are similar, with F1-scores of 0.90 and 0.92, respectively. These balanced and relatively high metrics indicate that the multidimensional temporal scattering characteristics of open-flame combustion aerosols are stable, and the neural network can reliably learn the unique optical evolution patterns of these two types of open-flame smoke.

Regarding interference aerosols, water vapor exhibited the best classification performance among all categories, with a precision of 0.99, a recall of 0.96, and an F1-score of 0.95. Water vapor consists of spherical liquid droplets, and its multi-angle scattering characteristics differ significantly from those of other solid particle aerosols. Therefore, it possesses a distinct and independent distribution region within the feature space. Dust samples exhibit the lowest metrics across all categories, with a precision of 0.81, a recall of 0.82, and an F1-score of 0.80. dust particles span a wide range of sizes, and their scattering responses tend to overlap with those of early-stage smoldering smoke, ultimately leading to reduced classification accuracy. The differentiated recognition results for various aerosol classes also reflect the distribution characteristics of different particles in the multi-channel temporal scattering space. The backbone network fully captures the differences in optical features resulting from variations in aerosol particle size, microscopic morphology, and concentration dynamics. Even when early-stage feature overlap exists between some samples, the network still successfully partitions the feature space for known classes.

### 3.2. Unknown Aerosol Identification Results Based on Out-of-Distribution Detection

**Figure 5:**
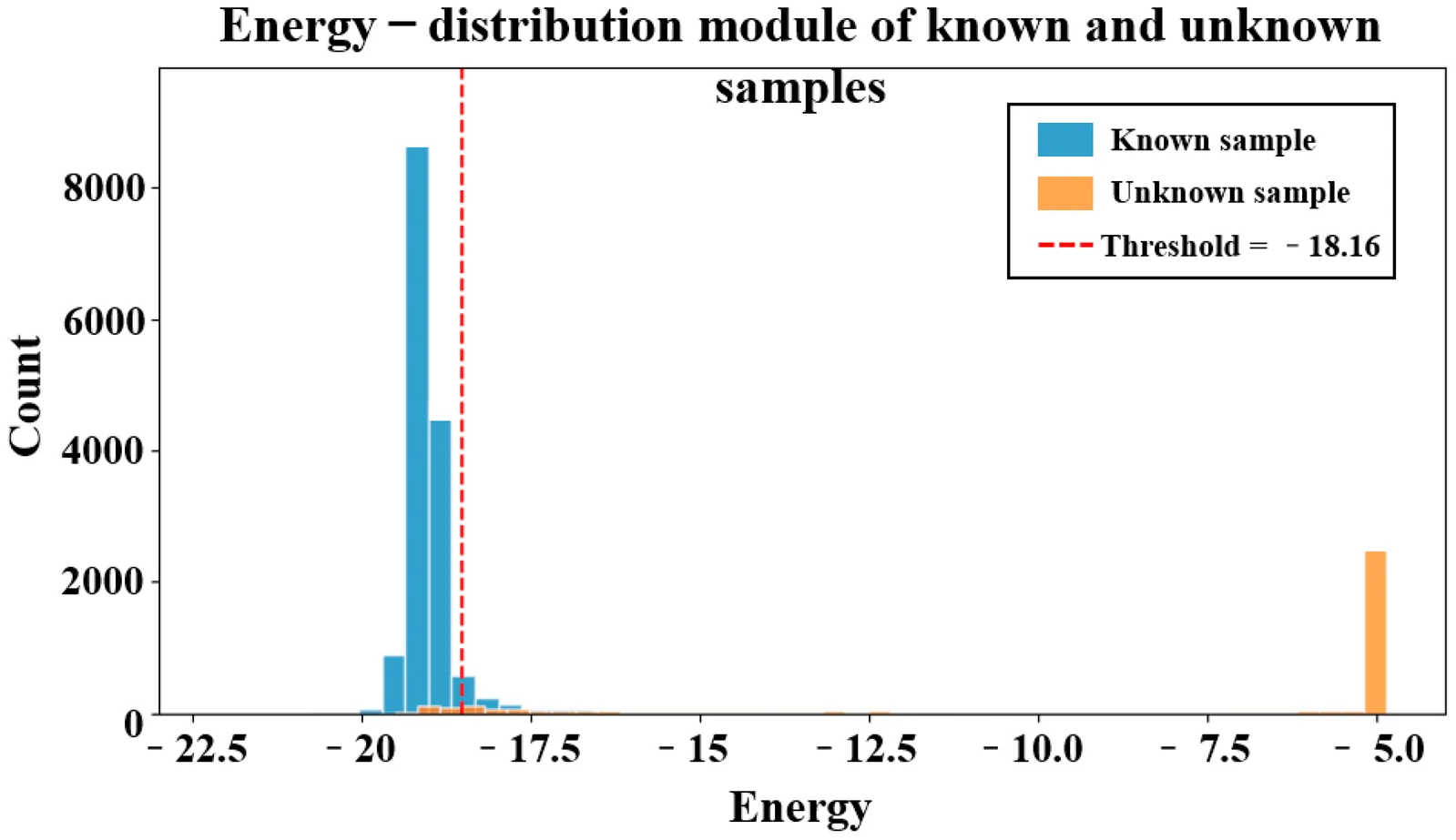
Energy-distribution module of known and unknown samples in the out-of-distribution detection stage.

The primary task of an out-of-distribution detection model is to map input samples to a scalar energy value, where a lower energy value indicates that the sample is more likely to originate from the training distribution. To evaluate the capability of FireOSR-Net to detect new aerosol classes not present in the training set, known and unknown aerosol samples from the test set were selected, fed into the out-of-distribution detection module, and their corresponding energy values and distribution characteristics were analyzed. The results are shown in Figure 5. Known aerosol samples are primarily concentrated in the lower-energy region, while unknown aerosol samples are generally shifted toward the higher-energy region. The two groups exhibit a distinct difference in distribution, indicating that the feature representations of unknown samples deviate more significantly from the known-class structure established during the training phase, which is consistent with the expected results.

The average energy of the known samples is −18.68, while that of the unknown samples is −8.32, a difference of 10.36. Based on the 95*_th_* percentile of the known sample energies, this work sets the open-set detection threshold at −18.16. The open-set threshold was determined using the validation set and was fixed before evaluating the held-out unknown categories. When the energy value of a test sample is greater than or equal to this threshold, the model classifies it as an unknown aerosol. This threshold lies between the main distributions of known and unknown samples, thereby reducing the risk of false rejections for known samples while maintaining detection sensitivity for unknown samples. However, this approach carries the risk that the distributions of the two sample classes may still overlap to some extent near the boundary. Therefore, the energy module does not assume that known and unknown samples can be completely separated. Instead, it uses thresholds to determine a classification boundary that balances false positives and false negatives. From the overall distribution, the average energy of known samples differs from the detection threshold by only 0.52, while the average energy of unknown samples is 9.84 higher than the detection threshold. This indicates that most samples can be reliably assigned to their respective regions. Furthermore, the pseudo-unknown feature generation strategy further expands the feature space beyond the known classes, enabling the energy module to learn a common representation that deviates from the known structure, thereby reducing the reliance on a limited number of true unknown samples at the open boundary. Therefore, it can be concluded that FireOSR-Net is capable of establishing an effective boundary for distinguishing between known and unknown samples based on the features of the Conv1d-LSTM backbone.

### 3.3. Fire Risk Prediction Results for Unknown Aerosols

**Figure 6:**
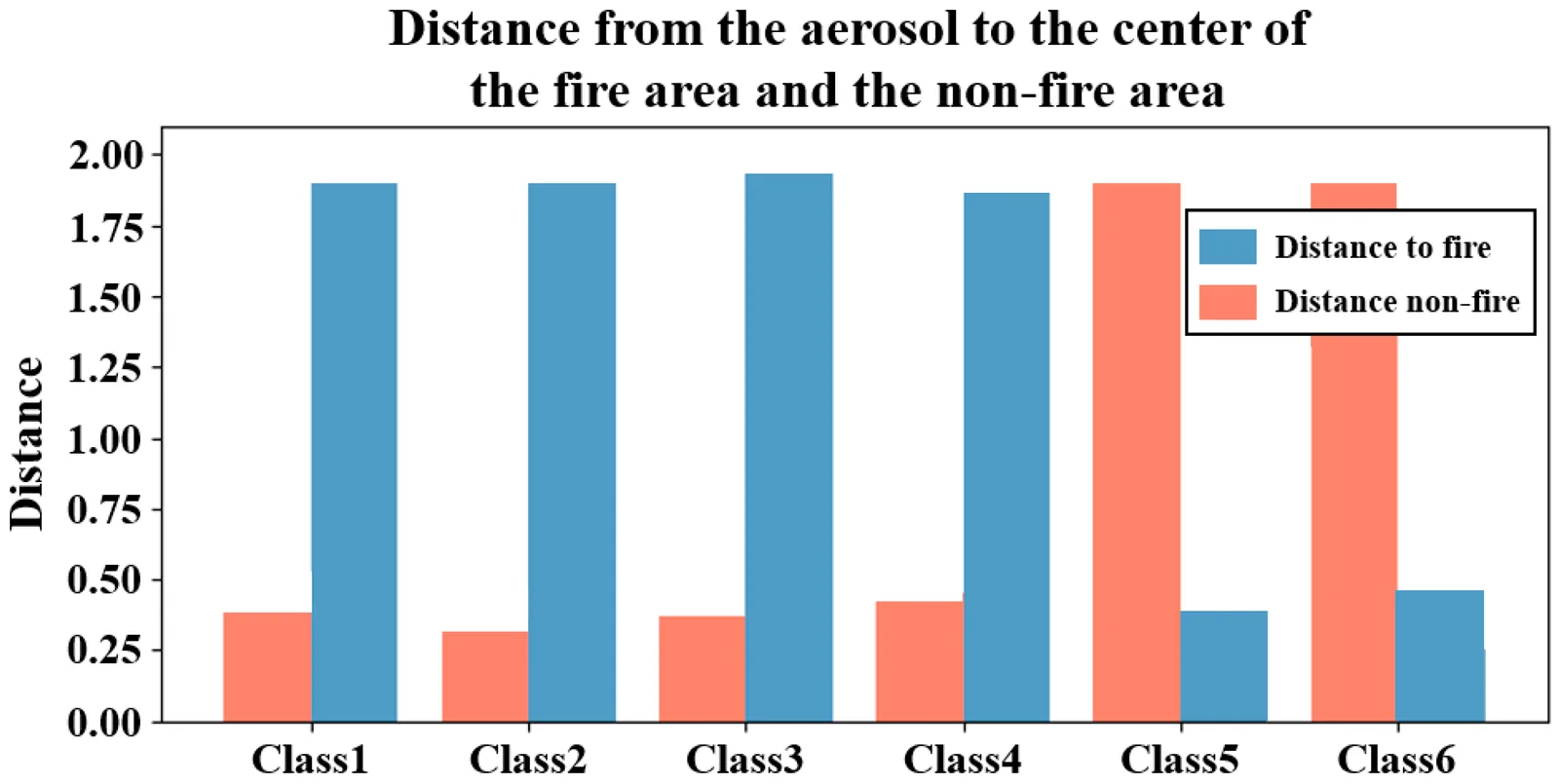
Prototype-domain separation learned by the prototype-based metric learning module. The bars show the distances from each class prototype to the fire semantic centroid and the non-fire semantic centroid, respectively.

After completing the open-boundary learning, fire and non-fire domains were further constructed in the prototype space. The results of the domain separation are shown in Figure 6, where the blue bars represent the distances of each type of aerosol to the center of the fire semantic domain, and the orange bars represent the distances of each type of aerosol to the center of the non-fire semantic domain, reflecting the overall separation between the two semantic domains. When an aerosol is closer to the center of the fire domain, it is more likely to belong to the fire smoke category. If it is closer to the center of the non-fire domain, it is more likely to belong to the interference aerosol category.

**Figure 7:**
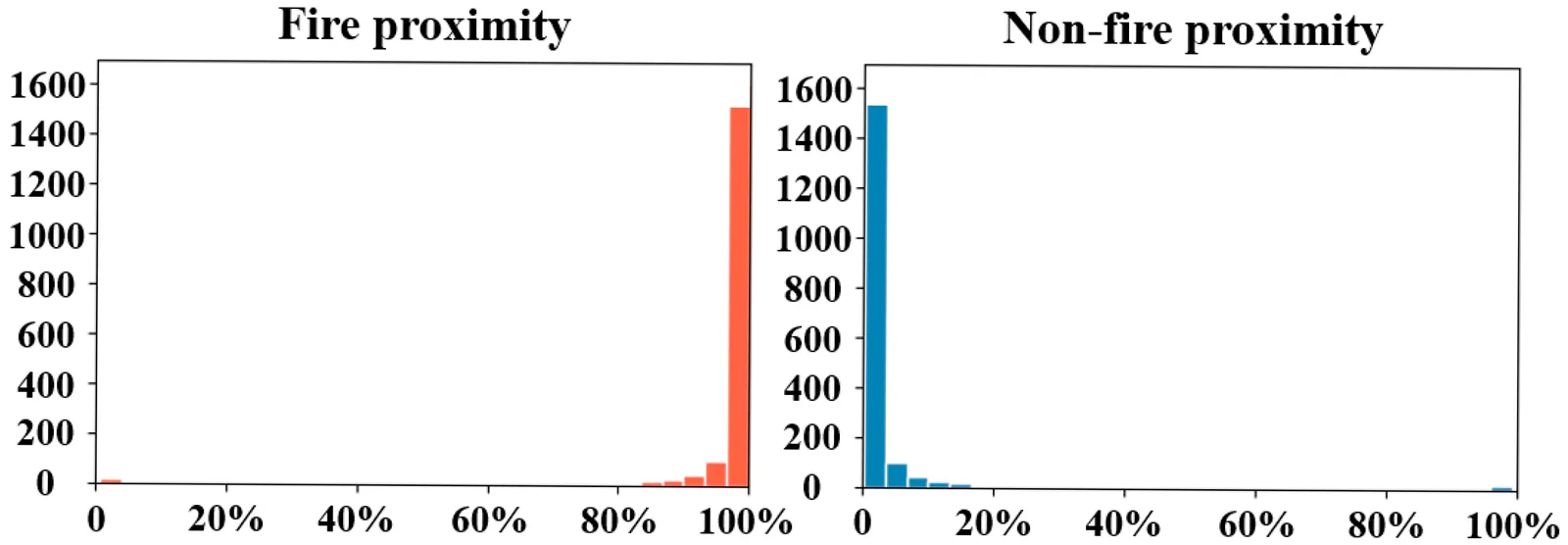
Histogram of proximity scores for unknown fire samples in the prototype space.

**Figure 8:**
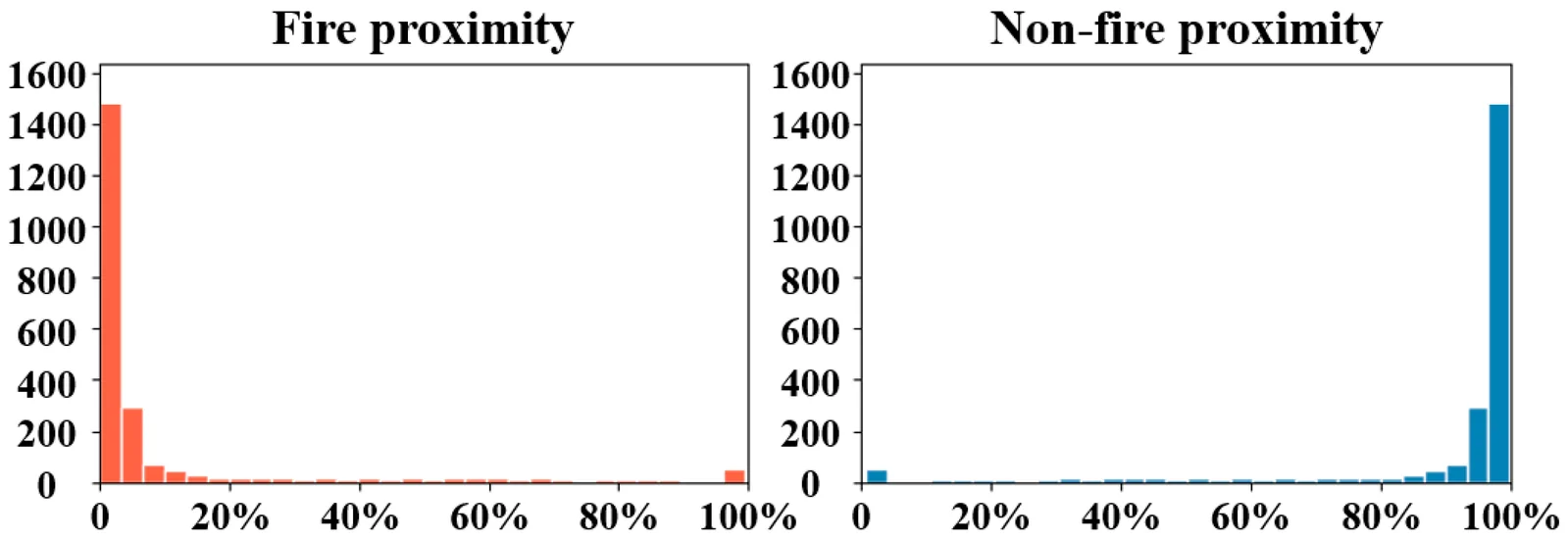
Histogram of proximity scores for unknown non-fire samples in the prototype space.

To evaluate fire risk classification performance of FireOSR-Net for unknown aerosol samples, this work maps unknown fire samples (i.e., smoldering wire) and unknown non-fire samples (i.e., oil fume) to the prototype embedding space and analyzes their proximity distributions, as shown in Figure 7 and Figure 8. Figure 7 shows that the fire proximity scores of the unknown fire samples are primarily concentrated in the high-value range. This indicates that, although these samples were excluded from training and identified as open-set inputs, they remain semantically close to fire-related domains. These distributions suggest that the prototype space preserves the tendency of unknown samples toward the fire domain. Figure 8 shows that the non-fire proximity of unknown non-fire samples is also primarily concentrated in the high-value range. This result indicates that even after classifying a sample as unknown, the model is still capable of further assessing its risk attributes based on its distance relationship to known prototypes.

Table 2 presents the statistical results for proximity and domain distance across the four sample groups: known fire, known non-fire, unknown fire, and unknown non-fire. The average fire proximity for the known fire samples was 93.23%, with average distances to the fire domain and non-fire domain of 0.15 and 1.86, respectively. The average non-fire proximity for known non-fire samples was 88.44%, with corresponding distances of 1.83 and 0.24. These results indicate that the prototype measurement module has established a relatively stable structure of fire and non-fire regions in the embedding space. Further examination of unknown samples reveals that the average fire proximity of unknown fire samples reaches 96.29%, with an average distance to the fire region of only 0.07 and an average distance to the non-fire region of 1.90. The average non-fire proximity for unknown non-fire samples reached 89.68%, with an average distance to the non-fire region of 0.25 and an average distance to the fire region of 1.79. This demonstrates that even when sample categories were not included in training, the model can still maintain a geometric distribution consistent with their true risk attributes in a higher-level semantic space. This result demonstrates that FireOSR-Net is capable of not only detecting unknown samples but also providing discriminative information on whether they are closer to fire smoke or non-fire interference, that is, it possesses the ability to extend from unknown sample detection to risk propensity analysis.

To further assess the generalization of FireOSR-Net beyond the two predefined unknown aerosol categories, we performed leave-one-category-out cross-validation on the expanded dataset. In each trial, one aerosol class was regarded as completely unseen unknown samples, and the remaining seven classes served as known samples for model training. All eight aerosol types took turns acting as unknown targets for open-set evaluation. High fire-domain similarity scores were obtained when the five fire-smoke categories were treated as unknown inputs: 0.92 for smoldering cotton smoke, 0.94 for beech smoke, 0.96 for polyurethane smoke, 0.90 for n-heptane smoke, and 0.96 for wire-smoldering smoke. These results indicate that the model captures shared optical and temporal features of fire smoke. It can correctly assign unseen fire aerosols to the fire domain even without corresponding training samples. For non-fire aerosols treated as unknown samples, non-fire domain similarity reached 0.91 for dust, 0.85 for water vapor, and 0.90 for oil fumes. The relatively lower score of water vapor originates from its weak and variable scattering signals under changing humidity. Even so, it still maintains effective domain discrimination. Consistent performance across all cross-validation groups proves that the model does not rely on several fixed unknown categories. FireOSR-Net learns stable feature differences between fire-smoke and non-fire-interference domains and shows favorable generalization for unseen aerosol types.

The fire-domain similarity score output by FireOSR-Net enables quantitative fire risk evaluation for smoke detectors. This metric can be used either as a primary indicator to trigger alarms with a properly calibrated threshold, or as complementary evidence fused with multi-sensor data for comprehensive risk assessment. Future work will optimize threshold settings and conduct field tests to support practical system integration.

### 3.4. Effect of the Temporal Feature Backbone

To quantify the effect of temporal feature extraction on model performance, we conducted a controlled comparison of the proposed Conv1D-LSTM backbone against three reference architectures: LSTM, gated recurrent unit (GRU), and temporal convolutional network (TCN). In each experiment, only the feature-extraction backbone was replaced; the remaining framework components and experimental conditions, including data partitioning, sliding-window configuration, optimization strategy, and evaluation protocol, were kept unchanged. This design enables performance differences to be examined with respect to backbone selection under a common evaluation setting.

As reported in Table 3, the Conv1D-LSTM backbone attained a closed-set average accuracy of 0.973, compared with 0.924, 0.929, and 0.942 for LSTM, GRU, and TCN, respectively. The corresponding absolute improvements were 4.9, 4.4, and 3.1 percentage points. For the open-set fire/non-fire proximity score, Conv1D-LSTM achieved 0.852, whereas LSTM, GRU, and TCN achieved 0.800, 0.732, and 0.806, respectively; the absolute improvements were therefore 5.2, 12.0, and 4.6 percentage points. Among the evaluated configurations, Conv1D-LSTM thus produced the highest value for both metrics.

The results indicate that, under the present dataset and evaluation protocol, the hybrid backbone is associated with improved closed-set classification and open-set proximity performance relative to the three reference backbones. A plausible explanation is that the convolutional layers capture local temporal patterns and inter-channel relationships in the scattering measurements, while the recurrent layer models dependencies across successive time steps. Their integration may provide a representation of both local signal variations and temporal evolution. This interpretation is consistent with the architecture, but is not independently established by the present comparison. Moreover, because repeated-run variability and statistical significance were not reported, the observed differences should be interpreted as descriptive rather than conclusive evidence of general superiority.

## 4. Conclusions

Accurate and reliable fire smoke detection is crucial for early fire warning. Unfortunately, in real-world monitoring scenarios, interference aerosols such as water vapor, dust, and oil fumes can easily trigger false alarms in photoelectric smoke detectors due to their optical scattering effects. In this work, we construct a four-channel photoelectric sensing system coupled with the proposed FireOSR-Net open-set recognition framework to mitigate false-alarm risks in aerosol-based fire smoke detection. The integrated framework performs known-aerosol classification, out-of-distribution sample detection and quantitative fire-risk propensity evaluation. Validation experiments were conducted using an 8-class aerosol dataset collected from a proprietary four-channel fire smoke detector. The results show that the F1-score for known samples ranges from 0.82 to 0.98, with an average fire proximity of 97.06% for unknown smoldering wire smoke samples and an average non-fire proximity of 89.41% for unknown oil fume samples. The experimental results demonstrate that, under the current experimental conditions, FireOSR-Net can simultaneously perform known aerosol classification, unknown sample detection, and fire risk propensity assessment, providing a viable technical approach for reducing false alarm rates in photoelectric smoke detectors and enhancing the interpretability of recognition results.

Nevertheless, FireOSR-Net was primarily validated under controlled laboratory conditions with a limited range of aerosol categories, and its ability to generalize across different scenarios requires further validation. In addition to the limitations of the dataset, this sensing system also has inherent hardware limitations. Contamination or blockage of the optical window can degrade signal quality and reduce the accuracy of open-set recognition. Furthermore, the optical path and photodetector determine the effective detection range. High aerosol concentrations can cause signal saturation, while low concentrations result in a poor signal-to-noise ratio. These issues cannot be fully resolved through algorithmic post-processing. Future work will expand the range of aerosol categories, conduct field tests, and implement the algorithm on hardware. To mitigate these hardware limitations, we will explore anti-contamination designs for the optical window and optimize the dynamic range of the sensing circuitry. Furthermore, we will investigate auxiliary signal calibration methods to further suppress hardware-related interference, thereby enabling practical deployment.

## Figures and Tables

**Table 1:** Closed-Set Classification Results for Aerosols of Known Classes.

Aerosol	Precision	Recall	F1-Score
Class1 (Cotton)	0.95	0.91	0.93
Class2 (Beech)	0.84	0.94	0.87
Class3 (Polyurethane)	0.94	0.88	0.90
Class4 (N-Heptane)	0.92	0.91	0.92
Class5 (Dust)	0.81	0.82	0.80
Class6 (Water vapor)	0.99	0.96	0.95

**Table 2:** Average Proximity and Distance of Aerosols to Fire and Non-Fire Domains.

Aerosol	Proximity to Fire (%)	Proximity to Non-Fire (%)	Distance to Fire	Distance to Non-Fire
Fire smoke	93.23	6.77	0.15	1.86
Non-fire reference aerosol	11.56	88.44	1.83	0.24
Unknown fire smoke	96.29	3.71	0.07	1.90
Unknown non-fire reference aerosol	10.32	89.68	1.79	0.25

**Table 3:** Comparison of temporal feature backbones using closed-set average accuracy and the open-set fire/non-fire proximity.

Backbone	Closed-Set Average Accuracy	Open-Set Fire/Non-Fire Proximity
LSTM	0.924	0.800
GRU	0.929	0.732
TCN	0.942	0.806
Conv1D-LSTM (Ours)	0.973	0.852

## Data Availability

The raw data supporting the conclusions of this article will be made available by the authors on request.
